# Heparin-binding protein measurement improves the prediction of myocardial injury-related cardiogenic shock

**DOI:** 10.1186/s12872-020-01406-3

**Published:** 2020-03-11

**Authors:** Tuo Pan, Guang-Feng Long, Cheng Chen, Hai-Tao Zhang, Jun-Xia Wang, Anshu Ahaskar, Hong-Bing Chen, Dong-Jin Wang

**Affiliations:** 1 Department of Cardio-Thoracic Surgery, Nanjing Drum Tower Hospital, The Affiliated Hospital of Nanjing University Medical School, Number 321 Zhongshan Road, Nanjing, 210008 Jiangsu China; 2 Department of clinical laboratory, Children’s Hospital of Nanjing Medical University, Nanjing, 210008 Jiangsu China; 3 Peking Union Medical College and Chinese Academy of Medical Sciences, Beijing, 100730 China

**Keywords:** Heparin-binding protein, Myocardial injury-related cardiogenic shock, Cardiopulmonary bypass

## Abstract

**Background:**

Heparin-binding protein (HBP), a potent inducer of increased vascular permeability, is a potentially useful biomarker for predicting outcomes in patients with postoperative myocardial injury-related cardiogenic shock (MIRCS). We aimed to evaluate and validate HBP as a prognostic biomarker for postoperative MIRCS.

**Methods:**

We performed a case-control study in 792 patients undergoing cardiac surgery from January 1, 2016, to August 1, 2019, including 172 patients with postoperative MIRCS and 620 age- and sex-matched controls. The association between HBP and MIRCS was determined by multivariate logistic regression analysis. Receiver operating characteristic curves (ROCs) with area under the curve (AUC) were performed to calculate the cut-off value, sensitivity and specificity. The association between HBP and cardiac troponin T (cTnT) was determined by multivariable linear regression analysis. Blood samples were drawn from the coronary sinus and arterial line of the cardiopulmonary bypass (CPB) before aortic cross-clamping (time point 1) and 5 min after aortic declamping (time point 2).

**Results:**

Before aortic cross-clamping, coronary sinus HBP (HBP_CS1_) showed no differences between the two groups. However, after declamping, the MIRCS group had a significantly higher sinus HBP level (HBP_CS2_) than did the control group. HBP_CS2_ predicted MIRCS with an AUC of 0.85 (95% CI: 0.81–0.89, cut-off: 220 ng/ml, sensitivity: 92% and specificity: 70%). After adjusting for confounding factors, we found that HBP was an independent risk factor for MIRCS (OR: 7.65, 95% CI: 4.86–12.06, *P* < 0.01) and was positively associated with cTnT (β > 0, *P* < 0.01).

**Conclusions:**

Elevated levels of coronary sinus HBP were useful biomarkers for predicting MIRCS after cardiac surgery.

## Background

The incidence of postcardiotomy cardiogenic shock is approximately 3–9% after cardiac surgical procedures [1, 2]. Despite initially successful resuscitation, mortality after refractory cardiogenic shock remains high, with more than 15% of patients not surviving to hospital discharge [2, 3]. Early recognition of warning signals and hence the correction of persistent inadequacy of cardiac function is therefore of particular importance, especially for patients with postoperative cardiogenic shock.

Heparin-binding protein (HBP), also called azurocidin or cationic antimicrobial protein of 37 kDa, is a multifunctional inflammatory mediator [4] with the ability to induce vascular leakage [5]. HBP is contained within the secretory and azurophilic granules of polymorphonuclear leukocytes and is rapidly released upon the adhesion of leukocytes to endothelial cells [4, 5]. The systemic inflammatory response following resuscitation from septic shock includes leukocyte activation, endothelial injury, and vascular response with vascular leakage; thus, an elevation in plasma levels of HBP is expected and might represent a potential prognostic biomarker [6]. In some clinical investigations, the release of HBP has been demonstrated in various infectious diseases caused by an array of septic shock [6–9]. Similar to septic shock, cardiogenic shock is also associated with leukocyte activation, endothelial injury and vascular hyperpermeability [10, 11]. A prospective multicentre observational study indicated that high plasma levels of HBP were associated with the severity of post-cardiac arrest and poor outcome [12]. Therefore, HBP should be theoretically related to cardiogenic shock after cardiac surgery. However, these study cohorts were composed of adults who had septic shock and/or cardiac arrest. No studies have reported the relationship between HBP and postoperative cardiogenic shock in patients who underwent cardiac surgery. It may be worthwhile to study HBP to determine whether this protein could be a useful predictor for poor outcomes in patients with postoperative cardiogenic shock after undergoing cardiac surgery. Additionally, postoperative cardiogenic shock may be associated with intraoperative myocardial injury (myocardial injury-related cardiogenic shock, MIRCS) [13]. Therefore, we designed this case-control study to confirm the hypothesis that high levels of HBP in patients who underwent open heart surgery would be associated with postoperative MIRCS.

## Methods

### Study design and settings

This study is a retrospective observational, convenience sample study of patients who developed cardiogenic shock after open heart surgery conducted at one Chinese academic centre. The study centre was a tertiary care academic medical centre (Nanjing Drum Tower Hospital, Nanjing, China). After receiving approval from the ethical committee of Nanjing Drum Tower Hospital, we implemented this study. Written informed consent was obtained from all patients before enrolment in this study. The inclusion criteria were as follows: all patients who received mitral valve replacement (MVR), aortic valve replacement (AVR), MVR + AVR and aortic surgery+AVR and were older than 18 years of age were enrolled in this study. The exclusion criteria were as follows: patients who did not have Swan-Ganz catheters before surgery and patients who had chronic obstructive pulmonary disease (COPD), coronary artery disease, left ventricular ejection fraction of < 35%, preoperative cardiogenic shock, New York Heart Association (NYHA) ≥ III, administration of antiplatelet agents in the previous 5 days, congenital heart disease, systemic glucocorticoid medication or perioperative glucocorticoid substitution, immunosuppressive medication, pregnancy, extracorporeal membrane oxygenation (ECMO) or intra-aortic balloon pump (IABP) initiation before surgery.

Postoperative cardiogenic shock may be associated with intraoperative myocardial injury. Therefore, we defined “MIRCS” as a composite of criteria related to myocardial injury. The criteria were consistent with any of the following conditions: ① impaired ventricular function (cardiac index at the end of surgery< 2.2 L/m2/min) [14, 15]; ② poor perfusion (arterial lactate level at the end of surgery> 5 mmol/L) [14]; ③ need for a large number of vasoactive-inotropic drugs (vasoactive-inotropic score at the end of surgery≥40) [14, 16] after the correction of all electrolytes and blood gas abnormalities while adjusting preload volume to optimal values; and ④ a cardiac troponin T (cTnT) level at 24 h after CPB ≥0.8 μg/L and an increase of more than 10% from 24 h after CPB to 48 h after CPB [17–20]. The diagnosis of MIRCS was confirmed when all 4 criteria were met. A total of 4671 patients underwent cardiac surgery in our hospital from January 1, 2016, to August 1, 2019. According to the MIRCS criteria, there were 172 patients with MIRCS, and the remaining 4499 patients without MIRCS were observed. To investigate whether HBP could predict MIRCS, we selected 172 patients with MIRCS and 620 age- and sex-matched controls for our study.

Controls were patients who had never had a diagnosis of MIRCS. We identified two to four controls for each case. The control subjects were selected for each case and matched for sex and age (± 2 years). Controls had to be alive with the same practice on the date that their matched case was diagnosed with MIRCS.

### Patient management, sample collection and biomarker assays

In the operating room, a Swan-Ganz catheter was preoperatively inserted into all patients. The cardiac index was measured using the Swan-Ganz catheter. All surgical operations were performed through a median sternotomy. The ascending aorta was cannulated with a patient size-appropriate cannula. Venous cannulations were chosen with separate cannulas in the superior and inferior vena cava. Based on an active clotting time of more than 480 s, heparin (200–400 U/kg) was used to achieve anticoagulation. The CPB circuit was primed with 1500–2000 ml of sodium lactated Ringer’s injection, 25–50 g of albumin and 2 g of magnesium sulfate injection (concentration: 10%). The initial volume of the antegrade cold blood cardioplegia solution (4:1 cardioplegia solution-to-blood ratio) was double the volume needed for the cessation of all cardiac electrical activity but never less than 1000 mL. Cardiac arrest was maintained by the retrograde infusion of 300 mL of blood cardioplegia solution (8:1 cardioplegia solution-to-blood ratio) every 20 min. The CPB flow was adjusted to a target mean arterial pressure (MAP) within 50 to 80 mmHg, mixed venous oxygen saturation over 70%, carbon dioxide pressure within 30 to 40 mmHg and temperature within 32 °C to 34 °C. At the end of CPB, protamine was initially used to reverse heparin at a 1:1 ratio such that the activated clotting time was returned to preoperative values. Additional doses of protamine may have been required if the activated clotting time was not appropriate. After CPB, vasoactive and inotropic agents, including dopamine (3–10 mg/kg/min), dobutamine (3–10 mg/kg/min), milrinone (0.3–0.6 mg/kg/min) and norepinephrine (0.02–0.10 mg/kg/min), either alone or combined, were given to maintain a MAP of at least 65 mmHg. Extra epinephrine (0.02–0.10 mg/kg min) or vasopressin (0.02–0.07 U/min) was added to vasoactive prescriptions if any cases in the vasopressin group had not reached a MAP of at least 65 mmHg. Extracorporeal membrane oxygenation (ECMO) would be initiatively implemented in the operating room if patients had refractory hypotension and met the following conditions [15]: ① long CPB time (CPB time of > 4 h); ② need for high-dose vasoactive drugs (vasoactive-inotropic score ≥ 40); ③ cardiac index of < 2.2 L/m^2^/min and MAP of < 60 mmHg; ④ arterial lactate level of > 5 mmol/L; and ⑤ failure in weaning from CPB.

A recent study reported that HBP was released from reperfused coronary circulation concomitantly with coronary neutrophil adhesion and myocardial injury [21]. This finding may suggest that compared with arterial HBP, the HBP drawn from CS may be a more sensitive method for the intraoperative assessment of myocardial injury. Therefore, blood samples were directly taken from the CS by surgeons and simultaneously drawn from the arterial line of the CPB by perfusionists. Moreover, the blood samples for research were drawn at two time points: ① time point 1: immediately before aortic cross-clamping; and ② time point 2: 5 min after aortic declamping [21]. It should be emphasized that the CS samples were obtained after ligaturing cannulations of the superior and inferior vena cava.

The samples were immediately placed into 5 ml tubes containing sodium citrate (BD vacutainer). The tubes were immediately centrifuged at 3000 rpm for 10 min, and separate aliquots of the plasma supernatants were stored at − 80 °C until analysis. Commercial enzyme-linked immunosorbent assay kits were used for measurements of HBP (Joinstar Biomedical Technology Co., LTD, Hangzhou, China). Additionally, peripheral arterial cTnT levels were measured at the clinical laboratory of Nanjing Drum Tower Hospital exactly 5 min after aortic declamping at the end of surgery (T0) and at 3 (T3), 24 (T24), 48 (T48), and 72 (T72) hours after surgery. Additionally, a part of the blood sample drawn from CS after 5 min of aortic declamping was also tested for cTnT (cTnT_CS2_). Finally, the vasoactive-inotropic score (VIS) [22] was determined using the following calculation: dopamine dose (μg/kg/min) + dobutamine dose (μg/kg/min) + [10 x milrinone dose (μg/kg/min)] + [100 x epinephrine dose (μg/kg/min)] + [10,000 x vasopressin dose (U/kg/min)] + [100 x norepinephrine dose (μg/kg/min)].

### Statistical analysis

The sample size was calculated by PASS 11.0 (NCSS, LLC, Kaysville, UT, USA) using logistic regression models, with *P* = 0.9, alpha = 0.05, and odds ratio = 1.74. The sample size was 164 (82 vs 82) according to the calculation. Therefore, 172 patients with MIRCS and 620 control subjects were recruited in this study. IBM SPSS statistical software (Statistics for Windows, version 25, IBM Corporation, Armonk, NY, USA) was used for analysis. Continuous variables were presented as the mean ± SD or, if appropriate, as the median with interquartile ranges (IQR). Discrete variables are depicted as frequencies (n, %). Normally distributed continuous variables were evaluated using Student’s t-test, or the Mann-Whitney U nonparametric method was used for non-normally distributed continuous variables. Continuous variables were determined to be normal in distribution by the Shapiro-Wilk test. Categorical data were compared using the chi-square test or Fisher’s exact test.

In this age- and sex-matched study, the association between HBP level and MIRCS was determined by multivariate logistic regression analysis. Covariates reaching statistical significance (*P* ≤ 0.10) in the univariate analysis and those considered clinically relevant were entered into a forward selection multivariable logistic regression model. Then, for each multivariable logistic model, collinearity and calibration were assessed respectively by the value of variance inflation factor (VIF) and Hosmer-Lemeshow test. Ultimately, there were 16 variables in the multivariate logistic regression analysis (Table 5). After adjusting for confounding factors related to HBP (diabetes mellitus, hypertension, atrial fibrillation, preoperative LVEF, EuroSCORE, intraoperative atrial fibrillation ablation, type of cardiac surgery and CPB time), multivariable linear regression analysis was used to investigate the correlation of HBP and cardiac troponin T. Receiver operator characteristic (ROC) curves were constructed to identify optimal cut-off values in association with outcomes. The optimal cut-off was defined as the value associated with the highest sum of sensitivity and specificity (Youden’s index). The two groups were analysed using repeated measures analysis of variance (ANOVA). Differences between the two groups were determined by repeated measures ANOVA with subsequent Bonferroni correction, with *P* < 0.05/n considered significant. A *P* value of < 0.05 was considered statistically significant.

## Results

**Fig. 1:**
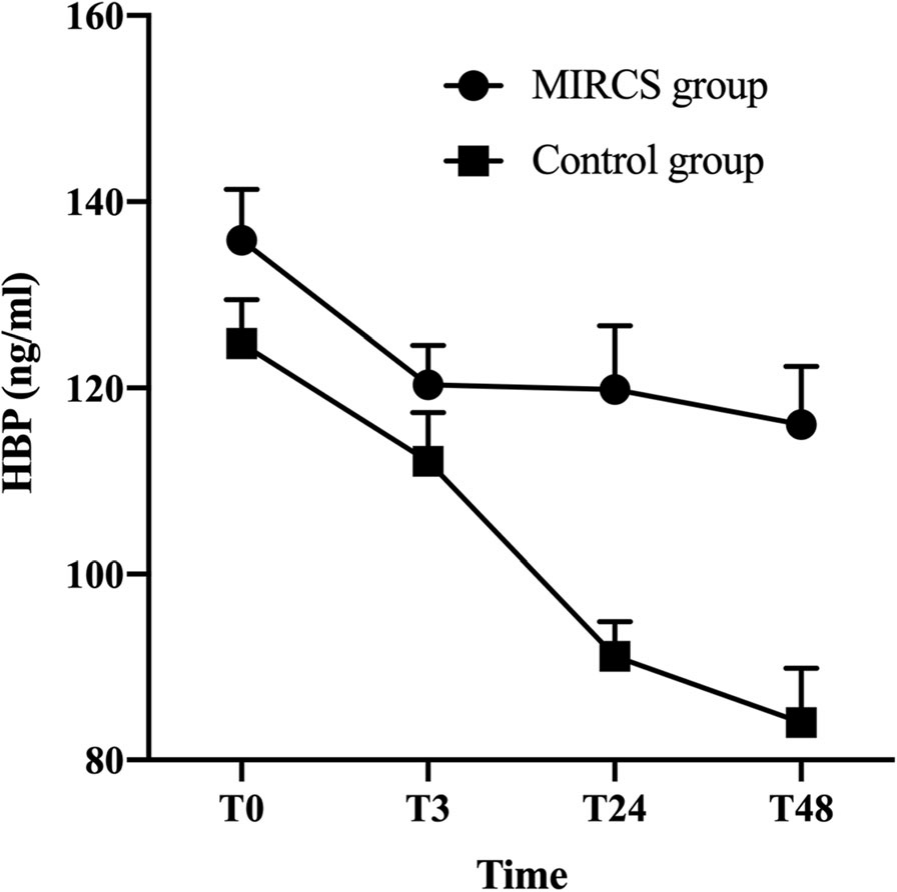
The MIRCS group had higher peripheral arterial HBP during the period from T0 to T48. T0: at the end of surgery, T3: at the 3rd hour after surgery, T24: at the 24th hour after surgery, T48: at the 48th hour after surgery

**Fig. 2:**
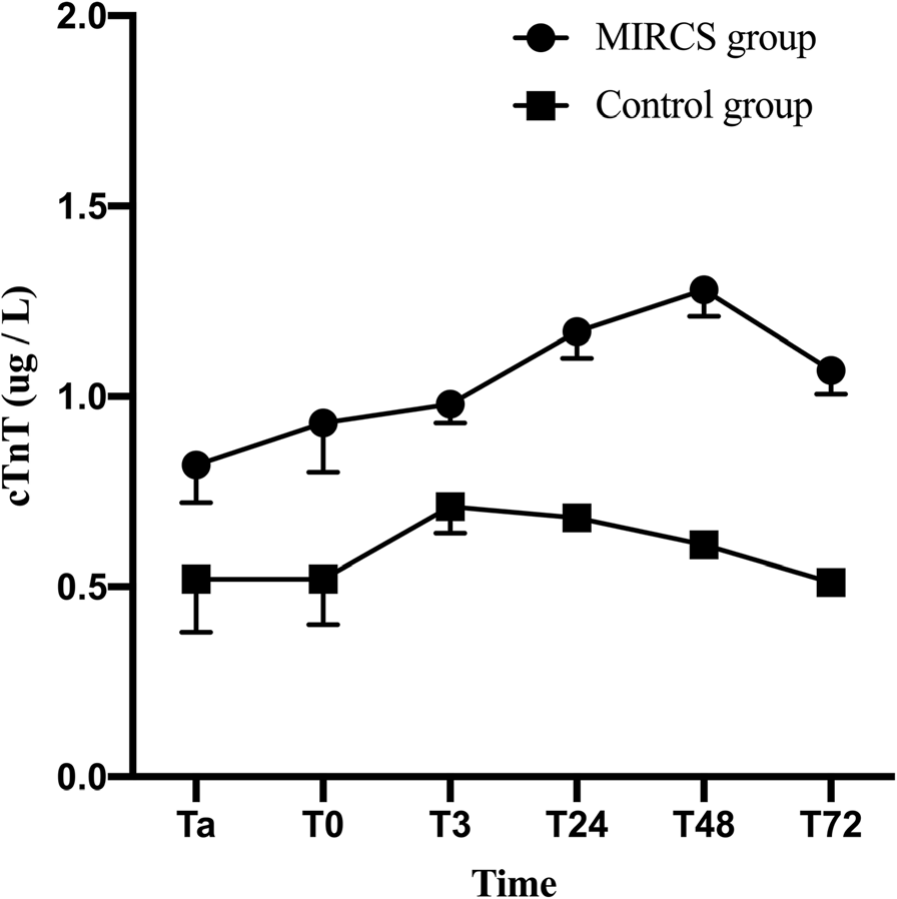
The MIRCS group had higher peripheral arterial cTnT during the period from T0 to T72. Ta: 5 min after aortic declamping, T0: at the end of surgery, T3: at the 3rd hour after surgery, T24: at the 24th hour after surgery, T48: at the 48th hour after surgery, T72: at the 72nd hour after surgery

There were 4671 patients who underwent valvular replacement and valvular + aortic surgery in our hospital from January 1, 2016, to August 1, 2019. A total of 792 patients met the inclusion and exclusion criteria, 172 of whom exhibited complicated postoperative MIRCS, and the remaining 620 age- and sex-matched patients had no postoperative MIRCS. No patients received urgent/emergency surgery in this study. Detailed demographic data are provided in Table 1. Patients with MIRCS had worse outcomes than patients in the control group. The outcomes in matched patients are presented in Table 2. HBP and cTnT levels and related variables for the MIRCS and control groups are presented in Table 3. The peripheral arterial HBP levels and cTnT levels increased later (Figs. 1 and 2; *P* < 0.01). After Bonferroni correction, the MIRCS group had higher peripheral HBP levels during the period from T24 to T48 (*P* < 0.05/4) and higher peripheral cTnT levels during the period from T0 to T72 (*P* < 0.05/6) compared with the control group. No significant differences in HBP_CS1_ and HBP_CPB1_ were noted between the two groups. However, the MIRCS group exhibited higher HBP_CS2_, HBP_CS2_ - HBP_CPB2_ and (HBP_CS2_ - HBP_CS1_)/HBP_CS1_, etc.

**Table 1:** Baseline and characteristics

Variable	Control (***n*** = 620)	MIRCS (***n*** = 172)	***P*** value
Age (year)	61.25 ± 13.16	60.45 ± 8.94	0.45
Gender (male)	282,45.48%	85,49.42%	0.39
Weight (kg)	64.72 ± 11.82	66.04 ± 14.23	0.22
Preoperative LVEF (%)	49.96 ± 10.60	50.45 ± 12.94	0.62
Preoperative LVDd (cm)	5.78 ± 0.85	5.79 ± 1.17	0.91
Preoperative cTnT (ug/L)	0.02 ± 0.02	0.02 ± 0.02	0.39
EuroSCORE	4.82 ± 3.45	4.39 ± 3.29	0.13
**Previous medical history**			
Myocardial infarction	3, 0.48%	0	0.36
Diabetes Mellitus (n,%)	45,7.26%	20,11.63%	0.07
Hypertension (n,%)	336,54.19%	79, 45.93%	0.02
Chronic Renal Failure (n,%)	0	3,1.74%	0.02
Liver diseases (n,%)	51, 8.23%	13,7.56%	0.75
Previous cardiac operation (n,%)	34,5.48%	6, 3.49%	0.27
Immunological diseases (n,%)	6, 0.97%	0	0.19
Atrial fibrillation (n,%)	334, 53.87%	68, 39.53%	< 0.01
Peripheral vascular diseases (n,%)	13, 2.10%	0	0.05
Blood products transfusion (n,%)	0	0	–
Heavily smoking (n,%)	72,11.61%	0	< 0.01
Excessive alcohol (n,%)	24, 3.87%	3,1.74%	0.24
**Type of cardiac surgery (n,%)**			0.02
AVR	21,3.39%	0	
MVR	340, 54.84%	82, 47.67%	
AVR + MVR	202, 32.58%	69, 40.12%	
Aortic operation + AVR	57, 9.19%	21, 12.21%	
Intraoperative AF ablation (n,%)	306, 49.35%	66, 38.37%	0.01
CPB time (minutes)	161.13 ± 74.83	169.86 ± 51.57	0.08
ACC time (minutes)	119.84 ± 65.42	127.51 ± 46.44	0.09

*cTnT* Serum cardiac troponin T, *MVR* Mitral valve replacement, *MIRCS* myocardial injury-related cardiogenic shock, *CPB* Cardiopulmonary Bypass, *AVR* Aortic valve replacement, *LVEF* Left Ventricular Ejection Fraction, *LVDd* Left ventricular end diastolic diameter

**Table 2:** Postoperative outcomes in age- and sex-matched patients

Variable	Control (***n*** = 620)	MIRCS (***n*** = 172)	***P*** value
**Adverse complications**			
Death (n, %)	0	10, 5.81%	< 0.01
ECMO use (n, %)	0	12, 6.98%	< 0.01
CRRT use (n, %)	95, 15.32%	95, 55.23%	< 0.01
Ventricular arrhythmias (n, %)	47, 7.58%	44, 25.58%	< 0.01
VIS > 40 more than 4 h (n, %)	149, 24.03%	172, 100%	< 0.01
**Other outcomes**			
Pneumonia (n, %)	8, 1.29%	45, 26.16%	< 0.01
Sepsis (n, %)	0	5, 2.91%	< 0.01
Re-intubation (n, %)	8, 1.29%	20, 11.63%	< 0.01
Re-operation (n, %)	8, 1.29%	14, 8.14%	< 0.01
MV time (hour)	9 (5, 16)	70 (65, 82)	< 0.01
Length of ICU stay (day)	3(2, 7)	19 (18, 23)	< 0.01

*MV* Mechanical Ventilation, *VIS* Vasoactive-inotropic Score, Median (interquartile range), *ECMO* Extracorporeal Membrane Oxygenation, *CRRT* Continuous Renal Replacement Therapy

**Table 3:** Perioperative variables in age- and sex-matched patients

Variables	Control (***n*** = 620)	MIRCS (***n*** = 172)	***P*** Value
**HBP before aortic cross-clamping**			
HBP_CS1_ (ng/ml)	56.21 ± 33.56	56.94 ± 26.37	0.79
HBP_CPB1_ (ng/ml)	40.65 ± 25.95	42.53 ± 28.97	0.41
**HBP after aortic declamping (5 min)**			
HBP_CS2_ (ng/ml)	163.13 ± 70.30	266.58 ± 114.24	< 0.01
HBP_CPB2_ (ng/ml)	151.97 ± 75.69	193.52 ± 69.50	< 0.01
**Related variables**			
HBP_CS1_ - HBP_CPB1_ (ng/ml)	10.02(5.10, 22.01)	7.01(0, 25.02)	0.52
HBP_CS2_ - HBP_CPB2_ (ng/ml)	11.01(−2.00, 22.50)	53.01(2.62, 84.75)	< 0.01
(HBP_CS2_ - HBP_CS1_) / HBP_CS1_ (%)	2.15(1.37, 3.23)	3.50(2.62, 5.39)	< 0.01
(HBP_CPB2_ - HBP_CPB1_) / HBP_CPB1_ (%)	3.03(1.91, 4.78)	4.36(2.18, 9.94)	< 0.01
HPB_(CS-CPB)_ ratio (%)	−0.14(−1.09, 1.22)	0.95(−0.59, 7.54)	< 0.01
CI wean from CPB (L/min·m^2^)	3.16 ± 0.44	1.85 ± 0.26	< 0.01
VIS wean from CPB	25.42 ± 15.97	51.02 ± 12.85	< 0.01
Lactate wean from CPB (mmol/L)	2.10(1.40, 2.50)	5.20 (5.10, 6.20)	< 0.01
cTnT_CS2_ (ug/L)	1.19(0.98, 1.75)	2.05(1.30, 2.76)	< 0.01
**Peripheral arterial cTnT (ug/L)**			
5 min after aortic declamping	0.52(0.19, 0.81)	0.82 (0.51, 1.50)	< 0.01
At the end of surgery (T0)	0.53(0.20, 0.83)	0.93 (0.55, 1.29)	< 0.01
At the 3rd hour after surgery (T3)	0.71(0.39, 0.96)	0.98 (0.98, 1.30)	< 0.01
At the 24th hour after surgery (T24)	0.68(0.56, 0.97)	1.17 (0.91, 1.61)	< 0.01
At the 48th hour after surgery (T48)	0.61 (0.47, 0.73)	1.28 (1.11, 1.63)	< 0.01
At the 72nd hour after surgery (T72)	0.51 (0.42, 0.75)	1.11 (0.92, 1.37)	< 0.01
**Peripheral arterial HBP (ng/ml)**			
At T0	125.22 ± 28.17	135.87 ± 32.67	< 0.01
At T3	112.11 ± 31.25	120.35 ± 25.35	0.03
At T24	91.17 ± 22.17	119.82 ± 40.87	< 0.01
At T48	84.04 ± 34.61	116.06 ± 35.25	< 0.01

*CI* Cardiac Index, *HBP*_*CS*_ Blood samples were obtained from coronary sinus, *Time point 1* Before aortic cross-clamping, *cTnT* Cardiac troponin T, Median (Interquartile range), *CPB* Cardiopulmonary bypass, *HBP*_*CPB*_ Blood samples were obtained from the arterial line of the CPB, *Time point 2* 5 min after aortic declamping, *VIS* Vasoactive-inotropic score, Mean ± SD, HPB_(CS-CPB)_ ratio = [(HBP_CS2_ - HBP_CPB2_)- (HBP_CS1_ - HBP_CPB1_)] / (HBP_CS1_ - HBP_CPB1_)

### Receiver operator characteristic curves and multivariate logistic regression analysis

**Fig. 3:**
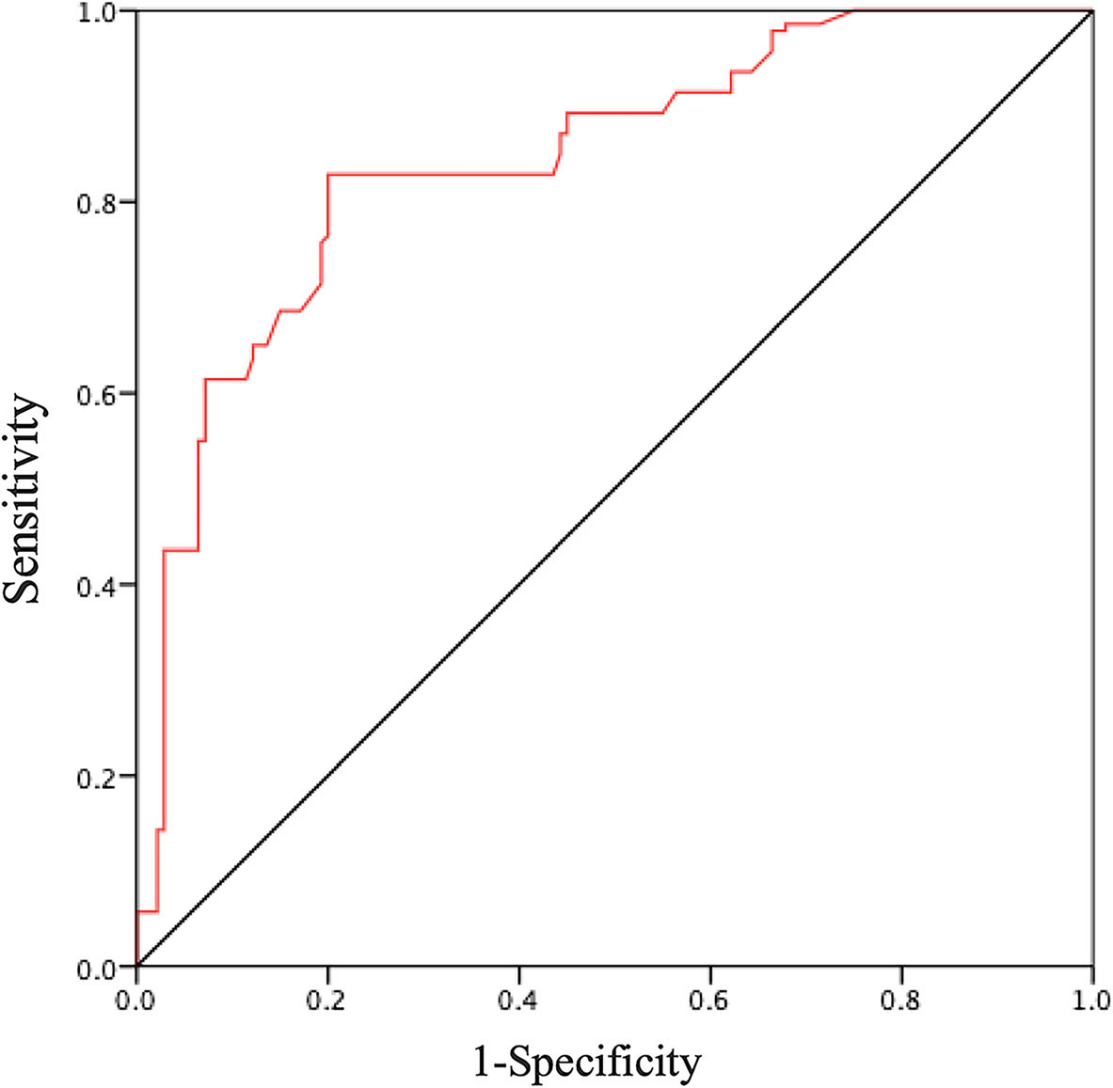
Receiver operator characteristic curves illustrating the abilities of heparin-binding protein to discriminate between control group and myocardial injury-related cardiogenic shock

The unadjusted ROC curves were established for predicting postoperative MIRCS of HBP_CS2_, HBP_CPB2_, HBP_CS2_ - HBP_CPB2_, cardiac index weaned from CPB, VIS weaned from CPB, and lactate weaned from CPB. (Table 4 and Fig. 3). The area under the ROC curve (AUC) values for peripheral arterial cTnT and HBP levels for the prediction of postoperative MIRCS are shown in Table 4. The AUC and best cut-off value for HBP_CS2_ were 0.85 and 220 ng/ml, respectively. This cut-off value resulted in a sensitivity of 92% and a specificity of 70%. According to cut-off values which demonstrated by ROC curves, we divided continuous variables into binary variables in multivariate logistic regression analysis (Table 5). In multivariate logistic regression analysis, HBP_CS_ (OR: 7.65, 95% CI: 4.86–12.06, *P* < 0.01) was independently associated with MIRCS.

**Table 4:** AUC for the predictors of MIRCS in age- and sex-matched patients

Variables	AUC	95%CI	Cut-off value	Sensitivity	Specificity
HBP_CS2_	0.85	0.81–0.89	220	0.92	0.70
HBP_CPB2_	0.71	0.64–0.77	140	0.88	0.46
HBP_CS2_ - HBP_CPB2_	0.85	0.81–0.90	30	0.71	0.99
(HBP_CS2_ - HBP_CS1_) / HBP_CS1_	0.75	0.69–0.81	3.38	0.65	0.81
(HBP_CPB2_ - HBP_CPB1_) / HBP_CPB1_	0.65	0.58–0.71	6.54	0.39	0.96
HPB_(CS-CPB)_ ratio	0.62	0.55–0.69	6.67	0.28	0.99
Peripheral arterial HBP at T0	0.64	0.59–0.68	114	0.85	0.44
Peripheral arterial HBP at T3	0.63	0.58–0.68	108	0.68	0.59
Peripheral arterial HBP at T24	0.71	0.67–0.75	87	0.84	0.53
Peripheral arterial HBP at T48	0.78	0.75–0.82	76	0.95	0.58
cTnT_CS2_	0.76	0.70–0.82	1.71	0.65	0.82
**Peripheral arterial cTnT**					
At 5 min after aortic declamping	0.70	0.64–0.77	0.42	0.92	0.41
At the end of surgery (T0)	0.75	0.69–0.81	0.78	0.64	0.70
At the 3rd hour after surgery (T3)	0.75	0.68–0.81	0.77	0.79	0.62
At the 24th hour after surgery (T24)	0.88	0.84–0.93	0.85	0.92	0.70
At the 48th hour after surgery (T48)	0.99	0.97–1.00	0.92	0.99	0.95
At the 72nd hour after surgery (T72)	0.96	0.94–0.98	0.76	0.99	0.82
Cardiac index wean from CPB	0.99	0.99–1.00	2.20	1.00	0.99
VIS wean from CPB	0.93	0.89–0.97	40	0.99	0.88
Lactate wean from CPB	0.92	0.88–0.97	4.45	0.99	0.92

*AUC* Area Under the Curve, *HBP*_*CS*_ Blood samples were obtained from coronary sinus, *Time point 1* Before aortic cross-clamping, *cTnT* Cardiac troponin T, *CI* Confidence interval, *HBP*_*CPB*_ Blood samples were obtained from the arterial line of the CPB, *Time point 2* 5 min after aortic declamping, *VIS* Vasoactive-inotropic score, HPB_(CS-CPB)_ ratio = [(HBP_CS2_ - HBP_CPB2_)- (HBP_CS1_ - HBP_CPB1_)] / (HBP_CS1_ - HBP_CPB1_)

**Table 5:** Multivariable logistic regression in age- and sex-matched patients

Variables	Odds ratio	95% CI	***P*** value
HBP_CS2_ > 220 ng/ml	7.65	4.86–12.06	< 0.01
**Peripheral arterial HBP**			
At the end of surgery (T0) > 114 ng/ml	1.50	0.93–2.42	0.10
At the 3rd hour after surgery (T3) > 108 ng/ml	1.23	0.79–1.92	0.35
At the 24th hour after surgery(T24) > 87 ng/ml	1.84	1.12–3.02	0.02
At the 48th hour after surgery(T48) > 76 ng/ml	1.54	1.00–2.37	0.05*
**Previous Medical History**			
Diabetes Mellitus	0.93	0.29–3.00	0.90
Hypertension	0.90	0.57–1.43	0.66
Atrial fibrillation	0.89	0.54–1.49	0.67
Preoperative LVEF	0.99	0.97–1.01	0.39
EuroSCORE	0.96	0.85–1.07	0.44
Intraoperative AF ablation	0.93	0.56–1.54	0.77
**Type of cardiac surgery**			
AVR	Reference		
MVR	0.88	0.46–1.71	0.71
AVR + MVR	1.81	0.83–3.94	0.14
Aortic operation + AVR	1.87	0.71–4.91	0.20
CPB time	0.99	0.99–1.00	0.37

*HBP*_*CS*_ Blood samples were obtained from coronary sinus, *MVR* Mitral valve replacement, *CPB* Cardiopulmonary bypass, *AVR* Aortic valve replacement

*P = 0.047

To further assess subjects who exhibited a correlation of HBP_CS2_ vs. cTnT, multivariate linear regression analysis was performed. After adjusting for confounding factors related to HBP (diabetes mellitus, hypertension, atrial fibrillation, heavy smoking, type of cardiac surgery and CPB time), there was a positive correlation between HBP_CS2_ and cTnT_CS2_ (B = 48.74, β = 0.43, *P* < 0.01) or peripheral arterial cTnT levels at 5 min after aortic declamping (B = 38.91, β = 0.21, *P* = 0.02), at T0 (B = 65.13, β = 0.32, *P* < 0.01), T3 (B = 100.38, β = 0.52, *P* < 0.01), T24 (B = 102.29, β = 0.48, *P* < 0.01), and T48 (B = 165.31, β = 0.84, *P* < 0.01) and T72 (B = 98.63, β = 0.40, *P* < 0.01).

## Discussion

Recently, published data demonstrated that HBP is associated with vascular leakage by capillary endothelium and breaking the cell barrier [5–9]. These study cohorts focused on adults who developed vascular leakage due to septic shock [6–9]. Such specific pathophysiological conditions of vascular leakage may be generally similar to those of coronary arterial endothelial injury resulting in MIRCS in patients after cardiac surgery. The key findings in our study were that coronary sinus HBP levels increased earlier than peripheral arterial HBP levels, and HBP was correlated with intra- and/or postoperative myocardial injury, providing an alternative means of predicting poor outcomes in patients with postoperative MIRCS.

### Relationship to previous studies

HBP is a granule protein mainly derived from neutrophils and is released from both secretory vesicles and azurophilic granules [5, 23]. Secretory vesicles release HBP rapidly upon the cross-linking of β2 integrins on the surface of neutrophils, while azurophilic granules release HBP more slowly [5, 24]. In vivo studies have shown that HBP is released not only upon neutrophil adhesion to endothelial cells but also when neutrophils are activated by circulating protein complexes formed by streptococcal M protein and fibrinogen, a virulence mechanism that was shown to induce severe organ damage [23, 25]. In some clinical studies, HBP was recently proposed as a biomarker for diagnosing septic shock [6–9]. Whole-body hypoperfusion during cardiogenic shock leads to endothelial activation and systemic inflammation [10, 11]. Therefore, HBP may be associated with cardiogenic shock. Pesonen et al. reported that HBP was released into reperfused coronary circulation at the time of coronary neutrophil adhesion and myocardial injury [21]. Ristagno et al. reported that elevated plasma heparin-binding protein is associated with early death after resuscitation from cardiac arrest [12]. These previous studies may confirm our hypothesis.

### Implications for practice

Our study demonstrated that HBP was increased in reperfused coronary circulation after CPB (HBP_CS2_ vs HBP_CS1_: 216.34 ± 110.36 ng/ml vs 56.69 ± 29.78 ng/ml, *P* < 0.01) and was associated with myocardial injury-related MIRCS in patients who underwent cardiac surgery. This finding may add evidence to the conclusion that HBP could act as a useful biomarker for the prediction of MIRCS. Theoretically, to predict the probability of myocardial injury-related MIRCS in patients who underwent open heart surgery, the HBP obtained from coronary circulation is more accurate than that obtained from systematic circulation. An observational study demonstrated that coronary sinus cTnT concentrations increased earlier and were higher than arterial concentrations during coronary artery surgery [26]. This finding may suggest that coronary sinus biomarkers may be a more sensitive method for the intraoperative assessment of myocardial injury. Our study indicated that cTnT_CS2_ was a better biomarker for predicting MIRCS than peripheral arterial cTnT at 5 min after aortic declamping (time point 2) and was correlated with HBP_CS2_. Moreover, in our study, HBP_CS2_ was positively correlated with peripheral arterial cTnT. HBP_CPB1_, HBP_CS1_, HBP_CBP2_, (HBP_CS2_ - HBP_CS1_)/HBP_CS1_, (HBP_CPB2_ - HBP_CPB1_)/HBP_CPB1_ and the HPB_(CS-CPB)_ ratio had low AUC values for predicting MIRCS. This result may confirm that HBP_CS2_ is not only an alternative biomarker for predicting MIRCS but also a candidate for predicting myocardial injury.

### Future directions

Considering the high morbidity and mortality associated with cardiogenic shock [2, 3], a better biomarker may assist physicians in managing the care of affected patients more effectively and improving outcomes. This is the first study investigating coronary sinus HBP in patients with MIRCS after open heart surgery. We found that coronary sinus HBP was a useful tool for predicting postoperative cardiogenic shock and myocardial injury. The presence of HBP_CS2_ > 220 ng/ml may be a useful complementary tool for the early identification of patients with postoperative cardiogenic shock and myocardial injury. It would improve outcomes in patients who underwent cardiac surgery.

### Study limitations

One limitation of this study was that it was conducted at a single institution as an observational study, which are prone to bias. Our study showed that peripheral arterial cTnT was positively correlated with HBP_CS2_. HBP is a biomarker for predicting inflammation [23–25]. Inflammation-sensitive proteins increase the incidence of ischaemic stroke and myocardial infarction [27]. This finding may be the reason peripheral arterial cTnT was correlated with HBP_CS2_. However, we had no evidence to directly confirm these results. Additionally, our study indicated that compared with HBP_CS2_, HBP_CS2_-HBP_CPB2_ had a noninferior power to predict postoperative MIRCS. However, there were some negative values in both groups. There may be potential statistical errors if HBP_CS2_-HBP_CPB2_ was regarded as a biomarker. In our study, patients with LVEF < 35% were excluded. These patients are prone to develop cardiogenic shock. However, they usually received CABG + valvular surgery or complicated valvular surgery, resulting in prolonged CPB time. The complicated operation and long CPB time would increase the incidence of severe inflammation. Furthermore, the cut-off of HBP might increase if these patients with LVEF< 35% were included. Therefore, we excluded these patients to avoid affecting the final results, although it may cause some statistical errors. Finally, the HBP may be suitable for the prediction of MIRCS in other heart operations, such as coronary artery bypass grafting (CABG). Currently, most heart surgery procedures in real-life clinical practice are CABG. However, perioperative cTnT may be difficult to control in CABG patients. This would add potential biases to our study. Moreover, the CABG procedures included off-pump CABG and on-pump CAGB. The off-pump CAGB was routinely implemented in our hospital. This means that coronary sinus HBP (HBPcs) could not be obtained in these patients. Therefore, our study did not include these patients. Thus, the implementation of the prognostic value of HBP should be assessed in future studies.

## Conclusion

Elevated levels of coronary sinus HBP were useful biomarkers for predicting MIRCS after cardiac surgery. Compared with peripheral arterial HBP, HBP collected from the coronary sinus is a more sensitive method for the intraoperative assessment of MIRCS.

## Data Availability

The datasets generated and/or analysed during the current study are not publicly available [some patients did not allow us to publish their medical records] but are available from the corresponding author upon reasonable request.

## Abbreviations

CPB: Cardiopulmonary bypass
CS: Coronary sinus
cTnT: Serum cardiac troponin T
ECMO: Extracorporeal membrane oxygenation
HBP: Heparin-binding protein
MIRCS: Myocardial injury-related cardiogenic shock
Time point 1: Before aortic cross-clamping
Time point 2: 5 min after aortic declamping
VIS: Vasoactive-inotropic score
